# Interventions to provide culturally-appropriate maternity care services: factors affecting implementation

**DOI:** 10.1186/s12884-017-1449-7

**Published:** 2017-08-31

**Authors:** Eleri Jones, Samantha R. Lattof, Ernestina Coast

**Affiliations:** 1 Department of Social Policy, London School of Economics and Political Science, London, UK; 2 Independent Consultant, Cardiff, UK

**Keywords:** Culture, Culturally-appropriate care, Pregnancy, Birth, Maternity care, Maternal health, Newborn health, Utilisation, Intervention, Implementation, Antenatal care

## Abstract

**Background:**

The World Health Organization recently made a recommendation supporting ‘culturally-appropriate’ maternity care services to improve maternal and newborn health. This recommendation results, in part, from a systematic review we conducted, which showed that interventions to provide culturally-appropriate maternity care have largely improved women’s use of skilled maternity care. Factors relating to the implementation of these interventions can have implications for their success. This paper examines stakeholders’ perspectives and experiences of these interventions, and facilitators and barriers to implementation; and concludes with how they relate to the effects of the interventions on care-seeking outcomes.

**Methods:**

We based our analysis on 15 papers included in the systematic review. To extract, collate and organise data on the context and conditions from each paper, we adapted the SURE (Supporting the Use of Research Evidence) framework that lists categories of factors that could influence implementation. We considered information from the background and discussion sections of papers included in the systematic review, as well as cost data and qualitative data when included.

**Results:**

Women’s and other stakeholders’ perspectives on the interventions were generally positive. Four key themes emerged in our analysis of facilitators and barriers to implementation. Firstly, interventions must consider broader economic, geographical and social factors that affect ethnic minority groups’ access to services, alongside providing culturally-appropriate care. Secondly, community participation is important in understanding problems with existing services and potential solutions from the community perspective, and in the development and implementation of interventions. Thirdly, respectful, person-centred care should be at the core of these interventions. Finally, cohesiveness is essential between the culturally-appropriate service and other health care providers encountered by women and their families along the continuum of care through pregnancy until after birth.

**Conclusion:**

Several important factors should be considered and addressed when implementing interventions to provide culturally-appropriate care. These factors reflect more general goals on the international agenda of improving access to skilled maternity care; providing high-quality, respectful care; and community participation.

## Background

Minority ethno-linguistic or religious groups often have poorer access to maternity care services than other populations [1, 2]; this poor access is linked to poorer maternal health outcomes [3, 4]. Health care providers that lack cultural competence, and differences in cultural practices and preferences between maternity care services and the communities they serve, can affect the decisions of women and their families on use of skilled maternity care [5–10]. The World Health Organization (WHO) recently made a recommendation supporting ‘culturally-appropriate’ maternity care services to improve maternal and newborn health [11]. Culturally-appropriate services, or providing care which takes account of the preferences and aspirations of individuals and the cultures of their communities, is an important component of quality of care [12].

We conducted a systematic review to examine evidence on the effects of interventions to provide culturally-appropriate maternity care for ethno-linguistic or religious groups on use of skilled care before, during, and after birth [13]. We considered interventions employing models of service delivery, service providers or service practices with the aim of providing culturally-appropriate care. Fifteen studies met our inclusion criteria, evaluating 14 different interventions [1, 5, 14–26]. Specific strategies included selecting health care providers who shared cultural and/or linguistic background with service users; employing cultural brokers, mediators or interpreters; providing staff training to improve cultural awareness; incorporating local birthing practices into service provision; adapting the physical or social setting in which a service is provided (e.g. equipping the delivery room with a rope and bench for vertical delivery, or including family in the room during the birth); and using participatory approaches. Some interventions focused on a single strategy while others adopted multiple strategies.

The review found that interventions to provide culturally-appropriate maternity care have largely improved women’s use of skilled maternity care [13]. Ten of 15 studies reported positive effects on at least one relevant care-seeking outcome, with most focusing on use of antenatal care (ANC). However, the contexts in which these interventions take place, and factors relating to their implementation, can influence their success. This paper examines factors that affected implementation of the 14 interventions included in our systematic review. We consider stakeholders’ perspectives and experiences of these interventions, and facilitators and barriers to implementation; and we conclude with how these factors relate to the interventions’ effects on care-seeking outcomes.

## Methods

This paper presents a secondary analysis of 15 studies included in our systematic review, described in detail elsewhere [13, 27]. The included studies measured the impact of an intervention to provide culturally-appropriate care for ethno-linguistic or religious groups on one of our outcomes of primary interest: birth with a skilled attendant, birth in a health facility, use of ANC, timing of first ANC visit, and postpartum care visits. To identify literature, we conducted systematic searches of ten electronic databases and two targeted websites [27]. We supplemented these searches with relevant literature identified in a related mapping [28]; hand-searches of the reference lists of included studies and related reviews; and suggestions from experts. We included studies published in English, French or Spanish between 1990 and 2014. We extracted data on the populations, interventions and study designs; and we conducted a quality assessment of each study using the Effective Public Health Practice Project quality assessment tool for quantitative studies [29].

For this secondary analysis of implementation factors, SL extracted data on contexts and conditions from each paper. EJ used a tool adapted from the SURE (Supporting the Use of Research Evidence) framework [30] to collate and organise these data according to a list of possible categories of factors that could influence implementation. Data on factors affecting implementation were largely provided in studies’ background and discussion sections. Some studies also included cost data or qualitative data.

## Results and discussion

Characteristics of the included studies, summarised in Table 1, are described in depth elsewhere [13]. The studies evaluated interventions in Australia (*n* = 5), the USA (*n* = 4), the UK (*n* = 2), Peru (*n* = 2), and Israel (*n* = 1). Most studies occurred in countries classified by the Organisation for Economic Co-operation and Development as high-income (*n* = 13); the exceptions were the two studies that took place in Peru, which is considered upper-middle-income [5, 20]. Most of the studies (*n* = 10) examined interventions targeting populations at the sub-national level (e.g. region, state, county, district), and the rest targeted populations at the local-level (e.g. village, neighbourhood). Indigenous women were the most common intervention recipients (*n* = 9), followed by ethno-linguistic minority groups in the USA or in the UK (*n* = 6). Several papers referred to overlapping characteristics, such as socioeconomic status, age and geographical location.

**Table 1:** Characteristics of the included studies

Study	Study Design	Setting	Description of Intervention	Reported Outcomes of Interest
Bilenko et al., 2007 [14]	Retrospective record review of ANC utilisation by pregnant women in two successive pregnancies, before and after the establishment of a local MCH clinic	ISRAEL, Negev Desert	A new maternal and child health clinic in desert areas for semi-nomadic Bedouin extended families living in tribal units, staffed by an Arabic-speaking Bedouin public health nurse	ANC
Gabrysch et al., 2009 [5]	Pre and post comparative study	PERU, Ayacucho rural Santillana district	A culturally-appropriate childbirth care model developed with Quechua communities and health professionals. Key features included a rope and bench for vertical delivery position, inclusion of family and TBAs, use of the Quechua language and health professionals that were respectful of culture	Skilled birth attendant, Facility birth
Jan et al., 2004 [15]	One qualitative component and two quantitative components (one economic and one that appeared similar to a retrospective cohort study)	AUSTRALIA, western Sydney	Daruk Aboriginal Medical Service, a community-controlled health service with a midwifery programme staffed by a team including an Aboriginal health worker. Features included regular ANC, transportation and home visits. Cultural awareness sessions were also provided for hospital staff	ANC
Jewell et al., 2000 [16]	Retrospective comparison of birth certificate data of infants born to project mothers and those born to non-project mothers	USA, Indiana	Minority health coalitions developed projects to increase access to early ANC for minority women through community outreach and addressing cultural factors that affect use of care. Strategies included use of minority professional and paraprofessional staff, social support, advocacy, and referrals for health education and transportation	ANC
Julnes, 1994 [17]	Retrospective comparison of women in the programme area in the intervention group with women who attended a clinic-based, multi-disciplinary programme and women who had no ANC, using a database constructed from monthly reports of births in the programme area, based on birth certificate information	USA, Norfolk, Virginia	Norfolk Resource Mothers Program - a community outreach programme using resource mothers or lay people, often sharing cultural background with the adolescents, to assist with non-medical dimensions of pregnancy and childcare, including getting ANC and acting as a liaison between the adolescents and public agencies	ANC
Kildea et al., 2012 [1]	A triangulation mixed method approach including mother and infant audit data, and routinely collected data from hospital databases	AUSTRALIA	Murri clinic – an antenatal clinic established in a tertiary hospital to provide antenatal services to Aboriginal and Torres Strait Islander women. Services include an Indigenous midwife and Indigenous liaison officers who helped families feel welcome, provided support for women in rural and remote areas and served as cultural brokers	ANC
Marsiglia et al., 2010 [18]	Randomised controlled trial	USA, Phoenix, Arizona	The Familias Sanas intervention was designed to bridge the cultural gap between Latinas and the health care system, and to reinforce among pregnant Latinas the importance of the postpartum visit. The intervention used bilingual, bicultural Prenatal Partners who served as cultural brokers. They showed participants how to navigate the health system and helped them improve communication with health care providers.	Postpartum care
Mason, 1990 [19]	Case-control	UK, Leicestershire, England	The Asian Mother and Baby Campaign was directed towards Asian women. Link workers, able to speak fluent English and at least one Asian language, worked alongside health professionals in the hospital and community setting as facilitators and interpreters while fulfilling an educative role.	ANC
McQuestion and Velazquez, 2006 [20]	An endline survey with mothers in the catchment areas of 29 treatment and 29 matched control facilities providing emergency obstetric care (EmOC). The probability of birth at the nearest public EmOC facility was modelled, conditional on whether the mother’s area participated in the programme, among other factors.	PERU, communities in high-risk *distritos* in 12 of 25 *departmentos*	Proyeto 2000 – a project to make emergency obstetric care services culturally acceptable, woman-friendly, and high-quality. Local birthing practices were incorporated into clinical protocols (specific features were not described). Qualitative data collected on mothers’ perceptions and preferences also informed a multimedia Safe Motherhood campaign; TBAs were trained; and facility staff engaged new community health committees.	Facility birth
Nel et al., 2003 [21]	Descriptive study (pre-post comparison)	AUSTRALIA, remote northern and western Queensland	Following consultations with health providers and Aboriginal communities, the programme included features such as a separate Indigenous medical centre managed by a community board and staffed by Indigenous people, home visits, provision of transportation and the involvement of family in ongoing care	ANC
NSW Health, 2005 [22]	Comparative study	AUSTRALIA, New South Wales	The NSW Aboriginal Maternal and Infant Health Strategy established community midwife and Aboriginal health worker teams to provide targeted, community-based, culturally-appropriate services for Aboriginal women in each area. State-wide training was introduced for these staff. Community development programmes were included to varying degrees across areas.	ANC
Panaretto et al., 2005 [23]	Prospective cohort study with a historical control group and a contemporary control group	AUSTRALIA, Townsville, north Queensland	Collaboration with Indigenous communities produced an integrated model of antenatal shared care, delivered from the community-controlled Townsville Aboriginal and Islander Health Service. Strategies included the use of Aboriginal health workers, continuity of care, and a family-friendly environment	ANC, Facility birth
Panaretto et al., 2007 [24]	Prospective cohort study of women attending the trial maternal child health programme compared with a historical control group	AUSTRALIA, Townsville, north Queensland	See Panaretto et al., 2005 (above)	ANC
Parsons et al., 1992 [25]	Retrospective study with control group	UK, Hackney, East London	The Multi-Ethnic Women’s Health Project – a health advocacy programme introduced at a hospital to meet the needs of non-English speaking women. Health advocates interpreted and mediated between service users and professionals to ensure an informed choice of health care	ANC, Care-seeking for complications or illness in women and newborns
Thompson et al., 1998 [26]	Retrospective study with control group	USA, rural Oregon	The Rural Oregon Minority Prenatal Program blended culturally-appropriate care with outreach by using bilingual and bicultural workers with strong links to their Mexican heritage, nursing case management and home visitation to facilitate access to ANC and community services	ANC, Care-seeking for complications or illness in women and newborns

Only one study used an experimental design, while all others used various forms of observational design. Four studies were assessed to be of moderate quality, with all others being of weak quality. Five papers included additional evaluation strands, most commonly interviews and/or surveys with service users and service providers or cost-effectiveness analyses [1, 5, 15, 22, 26]. Eight studies reported improvements in use and/or timing of ANC; one of three studies reported increases in birth at a health facility; and the one study that considered postpartum care reported a positive effect.

### Stakeholders’ perspectives and experiences of culturally-appropriate maternity care interventions

Since it was precisely the *in*appropriateness of existing services that interventions sought to address, improving acceptability and appropriateness according to stakeholders’ perspectives was fundamental. Culturally-appropriate interventions were designed based on empirical data, experience working with these communities and/or the input of communities through participatory approaches. Four of the included studies reported process evaluations that provided insight into the perspectives of community members. Each study that did report such data revealed largely positive views and experiences of the intervention [1, 5, 15, 22]. Gabrysch et al. [5] claimed that ‘simple changes such as respecting certain preferences or language or allowing the company of relatives can have a massive impact both on service satisfaction and use’ (p. 727). In their evaluation of a culturally-appropriate model for care at birth, developed with the participation of indigenous communities, 14 of 16 women were satisfied with the service, felt well-attended, would use it again and would recommend it to others. Women who used a community-controlled ANC service in Sydney, Australia, also reported a positive experience and emphasised improvements in relationships and trust, accessibility, flexibility, appropriateness of information, continuity of care, empowerment and family-centred care [15]. In another community-based intervention for Aboriginal women in Australia, women were positive about home visits, Aboriginal health workers, and assistance with transport [22]. Women also reported being generally satisfied with an indigenous antenatal clinic in Brisbane, Australia [1]. A much higher proportion of women ‘felt mostly understood and respected’ by staff in the intervention clinic (92%) than in other hospital locations, and they approved of the clinic location and care arrangements.

However, data also revealed some negative stakeholder perspectives. Jan et al. [15] found that stigma associated with a service specifically targeting an Aboriginal population appeared to prevent its use by some less vulnerable women. Stigma is one potential ethical implication that should be considered in any such intervention targeting specific groups, as well as the possibility that this may adversely affect use of skilled care for some women. Kildea et al.’s [1] interviews and surveys also indicated persistent problems with some aspects of the service, both from a community perspective and a health provider perspective, though interestingly these two groups did not always agree on what the problems were. For example, health providers and external stakeholders viewed the location of the clinic in a tertiary hospital to be problematic because of transport barriers; however, women reported that it was easy to access, though some said they would prefer a community-based location. Although making families feel welcome was a key element of the intervention, women reported that male partners were still uncomfortable with using services, particularly the waiting room. Both women and health providers identified broader problems that needed to be addressed. They reported that provision was too limited, delays too common, and arrangements too inflexible. They also reported problems with privacy that health workers believed hindered efforts to build relationships with service users.

### What factors affect implementation of culturally-appropriate maternity services?

Four key themes were prominent in our analysis of facilitators and barriers to implementation: accessibility; community participation; person-centred, respectful care; and cohesiveness between maternity services along the continuum of care through pregnancy until after birth.

### Accessibility

A complex range of factors affected use of skilled maternity care for targeted groups. Members of a cultural group might not use a service because they are too poor or because they live in a remote area [27, 10]. Studies highlighted the need to address broader access barriers alongside providing culturally-appropriate services. Poverty was a major issue and unless addressed, out-of pocket costs – direct or indirect – could discourage use even where culturally-appropriate services increased demand. Several studies noted context-specific issues with care financing that remained a barrier to uptake [18, 20, 26]. Physical access to maternity care services was also key; several populations targeted in these interventions lived in rural or remote areas [5, 14, 21, 26]. Populations in less remote areas did not necessarily have access to private transport or frequent, reliable and inexpensive public transport [1, 15]. Access was compounded by gender-based restrictions on women’s travel for some populations, such as semi-nomadic Bedouin women in Israel [14]. Many interventions adopted strategies to address physical access barriers alongside providing culturally-appropriate care. For example, two interventions transferred women living in particularly remote areas late in pregnancy to wait for birth in proximity to a maternity unit [5, 21]. Some interventions brought prenatal services closer to communities or adopted an outreach service [14–17, 22, 26]. Outreach often involved non-skilled workers who facilitated access to ANC, but women still needed to attend health facilities for skilled care. As discussed in the next section, several interventions using outreach models reported positive effects on use of ANC, but Thompson et al. [26] urged caution: they suspected that some women may have viewed these services as a substitute for ANC and suggested this as a possible reason for finding no effect on use or timing of ANC in their study. Several interventions provided transport services to health facilities [16, 21, 23], and an intervention with a Bedouin Arab population in Israel highlighted the need to ensure that transport provision itself is culturally-appropriate [14].

Women’s social circumstances have implications for access to care. Whether, or how, these circumstances were factored in was frequently cited as an enabler or barrier to interventions providing culturally-appropriate care. Women’s low levels of education or literacy; limited knowledge or experience of maternal health and health services; and a lack of social support were all described as challenges [14, 15, 17–19, 26]. Some interventions addressed these factors through the use of staff from the same cultural background as targeted populations to provide information, education and social support; to link communities with health services; and to facilitate access [14–19, 25, 26]. Childcare-related issues were compounded by transport problems and long waiting times [15, 26]. Some authors cited the provision of childcare as an enabler of their interventions [15, 16], and other authors deemed the lack of childcare provision to be an issue for future interventions to address [14].

### Community participation

Community participation was also a key strategy of several interventions reviewed, though the rationale, extent and type of participation varied widely. On the Spectrum of Participation, approaches ranged from consulting communities to shared leadership [31]. Among the studies in this review, dialogue with communities was seen to facilitate better understanding of problems with existing services and how they could be addressed to ensure that services met the needs of targeted populations [5, 14, 21, 26]. Dialogue between health providers and communities was seen as ‘crucial in building mutual respect’ [5]. The WHO recommends ongoing dialogue with communities as an essential component in defining the characteristics of culturally-appropriate, quality maternity care services that address the needs of women and incorporate their cultural preferences [13]. Mechanisms that ensure women’s voices are meaningfully included in these dialogues are also recommended. Several interventions also involved communities in the development, implementation, and/or monitoring of culturally-appropriate interventions. This deeper level of involvement gave communities ‘ownership’ and a stake in the interventions’ success [21]. In some interventions – particularly with Indigenous populations in Australia – this approach was operationalised through ‘community-controlled services’ [1, 21–23]. One intervention in Australia also established women’s reference groups to discuss, promote and support an enabling model of care, albeit with limited success due to low interest from community members [22]. State- and county-level grassroots minority health coalitions in the USA developed and implemented their own intervention, coordinating prenatal care projects to eliminate cultural barriers to care and to facilitate early entry into prenatal care [16]. Participatory approaches in maternal and newborn health interventions more generally have been reviewed elsewhere [13].

### Person-centred, respectful care

A pervasive barrier to uptake of care by target populations was poor interpersonal interaction with healthcare providers. Linguistic differences were a key barrier in many contexts [5, 19, 25, 26]. Women also reported that they faced unfriendly, insensitive and disrespectful interactions with health providers that were exacerbated by negative attitudes, discrimination and/or racism [1, 5, 15, 16, 25]. Poor interpersonal interactions resulted in anxiety and shame, and Jan et al. [15] noted that it ‘decreased [Aboriginal women’s] sense of self-worth and left them with feelings of inferiority’ (p. 18). Addressing interpersonal barriers was at the core of interventions to provide culturally-appropriate services. Employing staff members who shared linguistic and/or cultural backgrounds with target groups was the most common strategy [1, 14–19, 21–26]. Interventions also sought to build relationships and trust with target groups through friendly, non-judgmental, culturally-sensitive and respectful interactions [1, 15, 25, 26]; an empowering approach giving women choice [15, 18]; and continuity of care [1, 15, 22]. Studies reported that improvements in interpersonal interaction were at the forefront of facilitating their interventions.

Conversely, some studies described continuing problems with interpersonal care as barriers to implementation. A study in Peru indicated that building trust should receive more attention than it had already been afforded in their intervention [20]. Other studies noted that their interventions had been unable to surmount all challenges of interpersonal care. For example, a study in the UK was unable to hire female doctors to reduce target women’s discomfort with male doctors [19]. Studies also noted that problems with communication continued when the ‘cultural broker’ was not present [19, 26]. The latter point connects with the next and final theme.

### Cohesiveness along the continuum of care

Interventions frequently focused on one part of the continuum of care. For example, some interventions focused on making ANC services culturally-appropriate for specific groups of women, while care provided at birth to the same women was standard (i.e., not culturally adapted) [15]. Other interventions introduced an additional layer of ‘cultural brokers’, but the same health professionals continued to provide skilled care [18, 26]. These situations demand the building of effective partnerships and collaboration across providers or parts of the service. In particular, several studies highlighted problems when other providers that women came into contact with through pregnancy until after birth were not (as) committed to principles of cultural appropriateness. Jan et al. [15] sought to address this issue by providing cultural awareness sessions for local hospital staff. A lack of cohesiveness was acknowledged as a barrier to successful implementation of a prenatal nursing case management intervention for Mexican-American women in Oregon [26]. Staff had little control over other services their intervention sought to promote, which meant they were unable to ensure that women received culturally-appropriate care from other health care providers, despite efforts to ensure this within their own programme. Indeed, Thompson et al. [26] noted that women continued to face poor interpersonal care by doctors who ‘were not accustomed to the demands of this patient population and faced little prospect of financial reward’ (p. 87).

More generally, effective partnerships between the culturally-appropriate service and other providers that women and their families may encounter across the continuum of care from pregnancy until after birth is needed to ensure women receive a seamless service. Papers emphasised the need to forge links and coordinate with other service providers, and where possible to strive for information systems that prevent duplication [1, 15, 21]. An intervention in Peru improved links between service providers, community health workers and traditional birth attendants (TBAs), leading to a convergence of goals and improved referrals [5]. In contrast, Kildea et al. [1] found duplication between the culturally-appropriate service and mainstream services to be problematic in their intervention: ‘suboptimal communication between hospital and community-based providers contributed to operational inefficiencies […] In the absence of standardised protocols and reliable systems for information sharing, multi-agency maternity provision is not ideal and indeed, may impact negatively on the quality of care provided’ (p. 10).

## Conclusion

The studies include a range of interventions implemented with diverse populations in different contexts to provide culturally-appropriate services. While there are no one-size-fits-all rules to implementation, the findings and experiences of the 15 studies examined in this paper show that such interventions can make services more acceptable to the targeted populations and increase uptake of services. These implementation experiences highlight four key categories of enablers or barriers: accessibility; community participation; person-centred, respectful care; and cohesiveness along the continuum of care.

How do these enablers, barriers and stakeholder perspectives relate to the interventions’ effects on the care-seeking outcomes we reviewed? Table 2 illustrates the links between implementation factors and the reviewed studies’ reported effects on care-seeking outcomes. Three of five studies that included empirical data on community perspectives reported positive effects and high levels of satisfaction with the intervention [5, 15, 22]. The other two studies that found no improvements in uptake of services reported satisfaction with some elements of the intervention but not others [1, 26].

**Table 2:** Linking implementation factors with the systematic review outcomes

Studies from systematic review that report overall improvement in care-seeking outcomes		Findings from synthesis of factors influencing implementation	
Study	Setting	Important stakeholder perspectives critical to success	Implementation factors critical to successful outcomes
Bilenko et al., 2007 [14]	ISRAEL, Negev Desert	Recognition that women are often dependent on family members for transportation and that geographical barriers may further restrict access to medical services; recognition of female illiteracy	Establishment of maternal and child health clinics in desert areas serving a Bedouin Arab population living within 3 km, employment of an Arabic-speaking Bedouin public health nurse, the addition of a local Bedouin woman liaison worker
Gabrysch et al., 2009 [5]	PERU, Ayacucho rural Santillana district	Recognition of the importance of respecting traditional practices and including family in the birth process; acknowledgement of factors like low education levels, extreme poverty, previous conflict, and widespread female illiteracy; acknowledgement of limited transport options; recognition of inadequate communication between women and providers, either because the providers speak Spanish which is not understood by many or because provider rotation does not allow time to build trust; recognition that health professionals had treated women in unfriendly, brusque, and sometimes discriminatory ways	Hygiene procedures performed by the woman herself or family after explanations, provision of maternity waiting homes, inclusion of family, use of health providers who speak the Quechua language and are friendly and respectful of local culture, permitting women to wear their own clothes, changes to the delivery room setting (e.g. providing rope and bench to allow vertical crouching position, providing normal beds instead of gynaecological bed), integrating traditional Andean elements into the modern medical model (e.g. offering *rollete* if desired, placenta handed to family for burial), use of a participatory approach to ensure that services meet the local population’s needs
Jan et al., 2004 [15]	AUSTRALIA, western Sydney	Recognition that women will not return for services if they feel the male doctor is superior; recognition of inadequate communication between women and providers; recognition of the disempowering nature of hospital care for Aboriginal women and the inaccessibility of hospital clinics; acknowledgement that utilisation of services are influenced by factors like poor education, low income, high unemployment, and racial discrimination	Provision of transport service, short waiting times, provision of informal childcare, non-judgemental approach to providing care, cultural awareness sessions with local hospital staff, female general practitioners, Aboriginal health worker, provision of information in a way that suits women’s individual needs, assistance with infant feeding, flexible and proactive approach to seeing the client
Jewell et al., 2000 [16]	USA, Indiana	Recognition of factors influencing minority women’s poorer utilisation of early ANC than non-minority women (e.g. cultural insensitivity of providers, lack of encouragement to seek care, and the importance of advice from family and friends)	Staff helping women to work through the decision-making process on how to resolve barriers to their cultural beliefs and practices, staff providing advocacy for women if barriers occurred in navigating the health and social service systems, involvement of grassroots community-driven coalitions in the provision of culturally relevant care, provision of social support, provision of transport service, referrals to community services, health education, use of minority professional and paraprofessional staff, project monitoring by the minority health coalition boards, staff engaging in cultural brokering
Julnes, 1994 [17]	USA, Norfolk, Virginia	Acknowledgement that teenagers targeted by the intervention have limited social and financial support and may experience psychological barriers to ANC	Use of resource mothers (lay visitors) who often grew up in the same cultural milieu as the teenagers they serve (and were often teenage mothers themselves) and may be in a better position to provide empathy and social support, low cost of the intervention, encouragement of teenagers to seek ANC, provision of practical assistance to the teenagers and their families
Marsiglia et al., 2010 [18]	USA, Phoenix, Arizona	Acknowledgement of Latino spiritual and cultural beliefs related to health; recognition of the importance and influence of social support from family and friends; acknowledgement of cultural and linguistic influences that can become barriers between women and providers	Bilingual and bicultural Prenatal Partners who served as cultural brokers, active client outreach, improved communication between women and providers, patient-driven communication, encouragement of women to be active in their health decisions, education on prenatal care, development of a plan for ANC and postpartum visits
McQuestion and Velazquez, 2006 [20]	PERU, communities in high-risk *distritos* in 12 of 25 *departmentos*	Acknowledgement that utilisation of services is influenced by factors like poverty, social exclusion, and residing in a remote area; acknowledgement that facilities lack female caregivers; recognition of inadequate communication between women and providers, partly because the providers speak Spanish which is not understood by many; acknowledgement that reports of discrimination and mistreatment by health workers are commonplace	Extension of the Maternal and Child Health Insurance Program to cover most maternal and child health costs, including institutional delivery; emphasis on making services ‘woman-friendly’ (i.e. incorporation of local cultural beliefs and social norms into services, providing accessible and convenient facilities, offering high-quality services, guaranteeing confidentiality, respecting clients’ choices); use of mass media, health education and social mobilisation efforts promoting delivery in the nearest public emergency obstetric care facility
Nel et al., 2003 [21]	AUSTRALIA, remote northern and western Queensland	Recognition of the importance of extended family, acknowledgement that notes and test results must be shared between the medical centre and hospital facility, acknowledgement of women’s desire for continuity of care	Provision of transport service, ANC outreach visits, consultations with local Indigenous representatives to identify shortcomings and problems with ANC from an Indigenous perspective, inclusion of family at ANC consultations, use of Indigenous staff, patient tracking, seeing patients in a familiar setting, implementation of a shared care policy for doctors in the region
NSW Health, 2005 [22]	AUSTRALIA, New South Wales	Recognition that transport services are essential for access to health services and that in some places, access to ANC and midwifery services is non-existent; acknowledgement that some women are unable to afford fees for health care; acknowledgement that women value continuity of care and carer; recognition that some women chose not to utilise services due to the bureaucratic nature of mainstream public services (e.g. inflexible appointments, long wait times)	Statewide Training and Support Program for midwives and Aboriginal health workers, employment of an Aboriginal health worker or Aboriginal Health Education Officer, use of community development programs, taking a primary health care approach as opposed to a welfare model of care, basing services in the community where women could access care close to home in a familiar setting
Panaretto et al., 2005 [23]	AUSTRALIA, Townsville, north Queensland	Acknowledgement that the Australian Indigenous community had little evidence to guide ANC planning	Provision of transport service, family involvement, health care providers taking an integrated team approach, interventions for risk factors (e.g. smoking cessation, breastfeeding, testing for sexually transmitted infections, nutrition)
Panaretto et al., 2007 [24]	AUSTRALIA, Townsville, north Queensland	Health service providers and the Indigenous community working closely together to improve ANC	Provision of community-based and community-focused ANC, commitment to quality in service delivery, development of a sustainable health infrastructure, collaboration between health service providers and the Indigenous community to develop an integrated model of shared ANC

In contexts where physical access was recognised as a problem, studies that reported positive effects addressed this issue through either community-based services, provision of transport, or bringing women to health facilities to wait for the birth [5, 14–16, 21–24]. Two studies that did not find positive effects described persistent transport problems as a possible barrier to success [1, 26], though Thompson et al. sought to address this challenge through the intervention. Out-of-pocket costs were a greater barrier in some contexts than others due to differences in health care financing arrangements, but two studies that reported no improvements in care-seeking outcomes reported cost as a continuing barrier [20, 26]. These implementation factors therefore need to be addressed if care-seeking is to be improved.

Some level of community participation – at a minimum dialogue with communities – was an important component of several interventions reporting positive effects on uptake of care. Studies that found no improvements in uptake of care largely did not refer to community participation [1, 19, 25, 26].

Improving interpersonal interaction was reported as a fundamental element of almost all interventions to provide culturally-appropriate care, so this element did not necessarily distinguish interventions that reported improvements in care-seeking outcomes from those that did not. But two studies that did not find improvements reported that poor interpersonal interaction by other health care providers women encountered along the continuum of care through pregnancy until birth remained a barrier to women’s use of services [1, 26]. This finding relates to the challenge of ensuring cohesiveness across the continuum of care.

We acknowledge this paper’s limitations. First, the same limitations apply as those detailed for the systematic review [13, 27]. In particular, the possibility of publication bias means that we may not have captured the full range of implementation barriers and facilitators. Second, because our interest lay in how implementation factors relate to the success of interventions in increasing uptake of skilled maternity care, we considered only the interventions with impact evaluations included in the review. The literature on interventions excluded from our systematic review is broader geographically and describes additional interventions to provide culturally-appropriate care [27]. This broader literature underscores that efforts are being made in many settings to address and incorporate culture into maternity care. A review of this literature may provide further insight into implementation factors, but it was beyond the scope of our review. Third, a large portion of our data for this paper was drawn from the background and discussion sections of these papers, and this information was based on authors’ informed views on the reasons for their interventions’ success or lack of success. Only five studies reported empirical data on implementation factors, and they were not always reported in detail. The latter point demonstrates the need for future intervention studies to incorporate and report process evaluations that provide data and insight into pathways from interventions to outcomes.

In interventions such as these, the number of variables that may have implications for effectiveness is infinite. The limited scope of the current evidence base means that we do not currently know what works, in what context, and at what stage on the continuum of care through pregnancy until after birth. To develop such understanding, we need to increase the volume of studies evaluating these interventions, and for the reporting of these studies to include reflexive insights on their contexts, such as funding and politics. Only four studies mentioned factors related to funding and sustainability [5, 15, 17, 18]. The level of detail varied and was limited, with one study praising the programme’s ‘relatively low cost’ [17] and another noting that trained lay workers could easily replicate the ‘very cost-effective’ intervention [18]. Studies should also include deeper consideration of wider implications, particularly where specific groups are targeted with separate, tailored services. These studies also need better definitions and standardisation so that they contribute to a body of evidence rather than a disparate collection of studies [13]. This standardisation of definitions, evaluation, and reporting would promote our understanding of what differences in contexts or conditions explain differentials in success. A body of evidence is emerging for interventions with Indigenous populations in Australia, but it is still lacking on a global scale.

Many of the implementation factors we highlight in this paper overlap with elements that are recognised as important for improving global maternal and newborn health more generally, including addressing barriers to access, community participation, providing high-quality respectful care, and improving continuity of care. What makes them so pertinent in this review is that they are compounded by cultural and linguistic differences, and the targeted groups are among the most vulnerable in their respective societies. Thus, although the included studies are concentrated in high-income countries, the findings are likely to be relevant also to low- and middle-income countries, where a growing body of literature has described low quality of care and disrespect in maternity services [10, 32, 33].

If researchers, programmers and policymakers are going to address inequalities in maternity care and maternal health outcomes, an improved evidence base that moves beyond simple recommendations that ‘cultural factors should be taken into account’ is urgently needed. Substantive investment is also required to improve health managers’ and health providers’ abilities to interact with these groups and improve the responsiveness of services.

## Abbreviations

ANC: Antenatal care
EmOC: Emergency obstetric care
TBA: Traditional birth attendant
UK: United Kingdom
USA: United States of America
WHO: World Health Organization
